# Transactions of the American Institute

**Published:** 1889-12

**Authors:** 


Transactions of the American Institute. Session of 1889, Pemberton Dudley, M. D., Secretary.

If the reader of this volume be a homœopathist, or if he have any knowledge of Homœopathy, the idea must occur to him that the Institute devotes its time chiefly to subjects of less importance to the exclusion of those of vital interest. Such we believe would be the thoughts of a Hahnemann, or of a Bœnninghausen, or of a Hering. And surely something must be amiss in the work of a *homœopathic* society which would be condemned by such homœopaths as those we have named. We think there is not to-day one member of the Institute who believes the usual yearly work of his Society would be approved of by those great homœopaths we have named. Does this prove the Institute in the wrong, or would Hahnemann, Bœnninghausen, or Hering be wrong?

